# Geometric constraint of mechanosensing by modification of hydrogel thickness prevents stiffness-induced differentiation in bone marrow stromal cells

**DOI:** 10.1098/rsif.2024.0485

**Published:** 2024-10-02

**Authors:** Maria L. Hernandez-Miranda, Dichu Xu, Aya A. Ben Issa, David A. Johnston, Martin Browne, Richard B. Cook, Bram G. Sengers, Nicholas D. Evans

**Affiliations:** ^1^ Centre for Human Development, Stem Cells and Regenerative Medicine, Bone and Joint Research Group, Institute for Life Sciences, University of Southampton Faculty of Medicine, Southampton, UK; ^2^ Ningbo Institute of Technology, Beihang University, Ningbo 315800, People’s Republic of China; ^3^ Bioengineering Science Research Group, University of Southampton Faculty of Engineering and Physical Sciences, Southampton, UK; ^4^ Biomedical Imaging Unit, University of Southampton Faculty of Medicine, Southampton, UK

**Keywords:** stiffness, bone marrow stromal cells, differentiation, elastic modulus, thickness, osteogenic, hydrogel

## Abstract

Extracellular matrix (ECM) stiffness is fundamental in cell division, movement and differentiation. The stiffness that cells sense is determined not only by the elastic modulus of the ECM material but also by ECM geometry and cell density. We hypothesized that these factors would influence cell traction-induced matrix deformations and cellular differentiation in bone marrow stromal cells (BMSCs). To achieve this, we cultivated BMSCs on polyacrylamide hydrogels that varied in elastic modulus and geometry and measured cell spreading, cell-imparted matrix deformations and differentiation. At low cell density BMSCs spread to a greater extent on stiff compared with soft hydrogels, or on thin compared with thick hydrogels. Cell-imparted matrix deformations were greater on soft compared with stiff hydrogels or thick compared with thin hydrogels. There were no significant differences in osteogenic differentiation relative to hydrogel elastic modulus and thickness. However, increased cell density and/or prolonged culture significantly reduced matrix deformations on soft hydrogels to levels similar to those on stiff substrates. This suggests that at high cell densities cell traction-induced matrix displacements are reduced by both neighbouring cells and the constraint imposed by an underlying stiff support. This may explain observations of the lack of difference in osteogenic differentiation as a function of stiffness.

## 1. Introduction

The ability of cells to sense and respond to mechanical information from the extracellular matrix (ECM) is important for many biological processes [1]. ECM stiffness directs stem cell spreading, differentiation, migration and proliferation [2–4] and is now one of the most studied ECM mechanical properties. ECM stiffness has been shown to be of fundamental importance in specifying stem cell differentiation [5,6], and so the mechanical properties of tissues probably play a fundamental role in tissue development and regeneration.

Adherent cells sense the stiffness of their growth substratum by applying traction forces at their points of attachment and by sensing their dynamic displacement as a function of the applied force [7,8]. Generally, on stiff materials, the resistance to this applied force results in assembly of focal adhesions, F-actin and the generation of intracellular tension. This subsequently results in cell spreading. On soft materials, however, cells are unable to generate internal tension and as a result spread to a lesser degree [2]. Downstream signalling then regulates gene expression and directs cell migration, proliferation and differentiation in a process known as mechanotransduction [9].

It is important to note that the ECM stiffness that an adherent cell senses is not only dependent on the elastic modulus of the material. It may also be dependent on the dimensions of the material, the magnitude of the applied load and dynamic changes in material structure that may occur on time scales similar to those of cell-induced displacements. For small cells on large material structures, this is often negligible. However, it becomes important when, for example, the thickness of a soft material adherent to a stiff underlying support is reduced. Many studies of cell mechanobiology use ECM-modified polyacrylamide (PAAm) hydrogel as a cell culture substrate. PAAm is a material with linear elastic properties, often chosen as its stiffness can be controlled independently of other variables, and because deformations can be tracked using encapsulated fiduciary markers, rather than it reflecting the anisotropic and often viscoelastic materials in native ECM. For cell studies, this material is polymerized *in situ* and bound to a glass or plastic support for ease of handling. Several studies have shown that for a hydrogel with a defined, low elastic modulus, the cell begins to behave as if it is on a much stiffer material as the thickness (or depth) of the material decreases [7,10,11]. This is due to the constraint to cell-induced lateral hydrogel deformation imposed by the underlying stiff support—effectively the cell must induce a greater strain in a thin material than in a thick material for an identical lateral surface displacement [8]. This also becomes important on thicker materials when cells begin to act collectively. Trepat *et al.* noted that large colonies of Madin–Darby canine kidney (MDCK) cells were insensitive to substrate stiffness [12], and subsequently we found that this scaled with colony size [13], reflecting earlier work that demonstrated that groups of cells contract materials to a much greater degree than individual cells [14–16]. In effect, colonies exert much larger lateral tractions than individual cells, and thus ‘feel more deeply’ into materials. In addition, cells are able to sense and respond to the dynamic disturbances neighbouring cells impart on a common ECM, and cells can become mechanically coupled through the ECM material in the absence of any cell contact—cells can ‘feel each other’ through the material [17]. As groups of cells exert more strain on their materials than individual cells, these collective groups can feel each other at greater distances than isolated, individual cells. Taken together these data indicate that collective behaviour of cells and material dimensions must be considered when interpreting mechanobiological observations.

The effect of ECM stiffness on bone-derived stem cells has been widely studied due to its importance in skeletal repair and in tissue interactions with orthopaedic biomaterials. Increasing stiffness is generally correlated with increased osteogenic differentiation in cell populations containing putative skeletal stem cells in the published literature (e.g. bone marrow stromal cells (BMSCs) or mesenchymal stem cells (MSCs); reviewed in El-Rashidy *et al.* [18]). However, many of these studies examine differentiation at low cell density [5,19,20], and some studies conclude that matrix stiffness may not correlate with osteogenic differentiation, with some cell populations remaining insensitive [21–26]. Recently, Venugopal *et al*. showed the importance of cell density in differentiating human MSCs [27]. At lower cell densities, MSCs spread and divided significantly less on soft compared with stiff substrates. However, as cell density increased, cell spreading and division were increased on soft materials, and become similar regardless of the substrate stiffness. This indicates the likelihood that individual MSCs are able to sense the dynamic mechanical strains imparted on materials by neighbouring cells, which they interpret as an increase in stiffness. It remains unknown, however, how the ability of monolayers of cells to sense substrate stiffness may be impacted by changes in hydrogel dimensions.

In this study, we tested the hypothesis that reduced substrate thickness limits the ability of BMSCs to deform PAAm substrates and reduces their ability to mechanosense soft materials and differentiate accordingly, as measured by cell spreading and osteogenic activity. To achieve this we modulated hydrogel thickness, elastic modulus and cell density and compared cell differentiation and cell-induced hydrogel displacements.

## 2. Methods

### 2.1. Fabrication of polyacrylamide hydrogels

PAAm hydrogels were prepared according to the method of Pelham & Wang [2]. Glass coverslips (13 or 25 mm diameter; VWR International, Leicestershire, UK) were used as rigid support for the hydrogels, and were cleaned with tissue paper and functionalized with 0.1 M NaOH (Sigma-Aldrich, Gillingham, UK) on a plate heater at 80°C for 20 min. Next, coverslips were washed with distilled water, dried before covering the surface with 3-aminopropyltriethoxysilane (APES) at room temperature for 5 min (Sigma-Aldrich) and rinsed with distilled water. Dried coverslips were immersed for 30 min in 0.5% (v/v) glutaraldehyde (Sigma-Aldrich) in phosphate-buffered saline (PBS) (Sigma-Aldrich), washed and dried again. Hydrogels with different elastic moduli were prepared by varying the concentration of acrylamide–bisacrylamide. 12.5% (v/v) acrylamide, 1.5% (v/v) bisacrylamide and 85% (v/v) PBS for soft hydrogels and 20% (v/v) acrylamide, 24% (v/v) bisacrylamide and 55% (v/v) PBS for stiff hydrogels. The mixture was degassed for 15 min under a vacuum. Then 0.1% (v/v) of N, N, N′, N′-tetramethylethane-1,2-diamine (TEMED) and 1% (v/v) of solution of 10% (w/v) ammonium persulfate (APS) (Sigma-Aldrich) were added to the mixture and vortexed to initiate the polymerization. Specific mixture volumes were situated between a pre-treated coverslip and glass. Once the hydrogels polymerized, they were immersed in PBS for 10 min, carefully separated from the glass slide, placed on well plates with PBS and washed overnight at 4°C. Hydrogels were washed three times with new PBS, covered with sulfosuccinimidyl 6(4-azido-2-nitrophenyl-amino) hexanoate (sulfo-SANPAH) (ThermoFisher Scientific, Loughborough, UK) 0.5 mg ml^−1^ in 4-(2-hydroxyethyl)-1-piperazineethanesulfonic acid (HEPES) and exposed to UV light (Chromato-vue TM−20, UVP transilluminator, 240 V) for 25 min. Later, the hydrogels were washed three times with HEPES 50 mM pH 8.5, and 0.1 mg ml^−1^ collagen solution type I (Sigma-Aldrich) was added to cover the hydrogels before incubating overnight at 4°C. For hydrogels containing fiduciary markers, fluorospheres at a concentration of 1% (v/v) (ThermoFisher Scientific) of 0.5 µm diameter were included in the PAAm mixture before polymerization and sonicated before use. For measurements of hydrogel thickness, allylamine (Sigma-Aldrich) was added at 0.196% v/v to the acrylamide-bis-acrylamide mixture before polymerization [13]. Once the PAAm hydrogels polymerized, hydrogels were incubated in Alexa Fluor 568 (Thermo Fisher Scientific) 1 mg ml^−1^ (1 : 50) at room temperature for 3 h before washing three times with PBS 1×.

### 2.2. Measurements of hydrogel thickness

Hydrogel thickness was measured by confocal microscopy (Leica TCS SP5, Leica, Cambridge, UK). Soft and stiff PAAm hydrogels of different thicknesses (three samples per condition) on 13 mm glass coverslips were placed upside-down on a thin glass slide and immersed in 1× PBS. Hydrogels were imaged at 20× magnification and 2 or 10 µm *z*-stacks from top to bottom. The fluorescent intensity profiles were obtained and analysed using the Leica Software (LAS X Core Offline) to calculate the thickness (*z*-value). The images were used to quantify manually the number of wrinkles on the hydrogel surface.

### 2.3. Measurements of hydrogel elastic modulus

Soft and stiff PAAm hydrogels with different thicknesses were fabricated as previously described, and stiffness was measured using a nanoindenter (NanoTest Vantage system; MicroMaterials Ltd, Wrexham) as described in Xu *et al.* [28]. In brief, the samples immersed in PBS solution were tested using a spherical diamond tip (500 µm). Nanoindentation was carried out in load control to various maximum loads (10–850 μN, minimum load step: 2 μN) to obtain the corrected elastic modulus, which is independent of substrate thickness and accounts for poroelastic effects, as described in Xu *et al.* [28]. The indentation depth/hydrogel thickness ratios (δ/h) varied between 0.01 and 0.5, with the indents spaced apart by 250 µm. The maximum load was applied for 120 s at rates of 1 µN s^−1^ for loading and 5 µN s^−1^ for unloading at 20 ± 1°C.

### 2.4. Cell culture

BMSCs were previously isolated from human bone marrow samples obtained from the Spire Southampton Hospital and the Southampton General Hospital under local ethical approval. As previously described [29], Stro-1+BMSCs (passage 1–5) were grown in α-MEM media with 10% v/v fetal bovine serum (FBS) and 100 µg ml^−1^ penicillin/streptomycin on tissue culture polystyrene flasks and incubated at 37°C. The medium was changed every 2–3 days. BMSCs obtained in this manner have been characterized previously for proliferation and trilineage potential [30]. For experiments, cells were subcultured from subconfluent proliferative cells.

### 2.5. Actin, nuclei and vinculin staining

Vinculin was stained to identify focal adhesions in Stro-1+BMSCs by immunocytochemistry. Cells were fixed in 4% (w/v) paraformaldehyde (PFA) for 20 min at room temperature, rinsed with PBS 1× and permeabilized with 0.5% (v/v) Triton, X-100 in PBS for 30 min at the same temperature. After washing three times with PBS 1×, cells were incubated in 0.1% (w/v) bovine serum albumin (BSA) in PBS at 4°C for 2 h. Next, cells were incubated with the primary vinculin rabbit anti-human, mouse, polyclonal antibody (ThermoFisher Scientific) at 2 µg ml^−1^ (final concentration) in 0.1% (w/v) BSA in PBS overnight at room temperature. Cells were washed with 0.1% (w/v) BSA in PBS three times and incubated with the goat anti-rabbit IgG (H + L) highly cross-adsorbed secondary antibody, Alexa Fluor Plus 594 at 10 µg ml^−1^ (final concentration) for 1 h at room temperature. FITC-conjugated phalloidin (ThermoFisher Scientific) (1 : 1000) was added to stain actin fibres and incubated for 30 min at room temperature (foil covered) and finally rinsed three times with PBS 1×. Thirdly, cells were stained with DAPI for 5 min at room temperature to stain nuclei and finally rinsed with PBS twice. Cells were imaged using a Nikon Ti inverted microscope and Leica confocal microscope with filters for DAPI (em ʎ = 350 ± 50 nm; ex ʎ = 460 ± 50 nm); eGFP (em ʎ = 470 ± 40 nm; ex ʎ = 525 ± 50 nm); Cy3 (em ʎ = 545 ± 25 nm; ex ʎ = 605 ± 70 nm) and Cy5 (em ʎ = 620 ± 60 nm; ex ʎ = 700 ± 75 nm) and merged using ImageJ/FIJI v. 2.9.0 free software.

### 2.6. Cell spreading area quantification

BMSCs were plated on PAAm hydrogels and tissue culture polystyrene, incubated for 24 h at 37°C and placed under a Nikon Ti inverted microscope. Five microscopic fields of each soft and stiff hydrogel of different thicknesses (*n* = 3) and phase contrast images were imaged at 10× magnification. The pictures were analysed using the FIJI software by drawing around the cell periphery using the wand (tracing) tool and by using the analysis menu for quantification. The cell area was measured using the analysis menu. The number of cells varied depending on the hydrogel elastic modulus and thickness.

### 2.7. Time-lapse imaging and digital image correlation

Time-lapse imaging was used for displacement microscopy studies to determine how cells perceive the rigidity of materials in different conditions, as described previously [13]. Briefly, cells plated on soft and stiff PAAm hydrogels with different thicknesses were photographed every 5 min for 24 h starting at different time points (days 1, 7, 10, 14, week 7, if applied) using a Nikon Eclipse Ti inverted microscope. The fluorescent images were acquired using the Cy3 channel (em ʎ = 545 ± 25 nm; ex ʎ = 605 ± 70 nm) and were saved as an ND2 file (Nikon, UK). The images were extracted from the ND2 file and uploaded into a MATLAB algorithm developed by Zarkoob *et al.* [14] using digital image correlation to track displacements. Firstly, a grid (10 × 8) was drawn to yield 99 nodes for the analysis. The tracking parameters were kernel size = 31; subpixel size = 9; smoothness = 5; maxMove = 6; smoothGrid = 25. MATLAB (The Math Works R2017a, Natick, MA, USA) was then used to quantify the total cumulative displacements from the first image taken at 5 min (image 1) to the following taken every hour (image 2, 3, ... , 24, etc.) in each node, by registering and adding up the changes in displacements between images taken 1 h apart. The cumulative displacement data from the 99 nodes in three hydrogel triplicates were used to calculate the 90th percentiles and standard deviation at each hourly time point. The data were plotted in GraphPad Prism v. 10 as XY graphs, and the average of the 90th percentile from approximately 8 to 24 h as grouped graphs.

**Figure 1.**
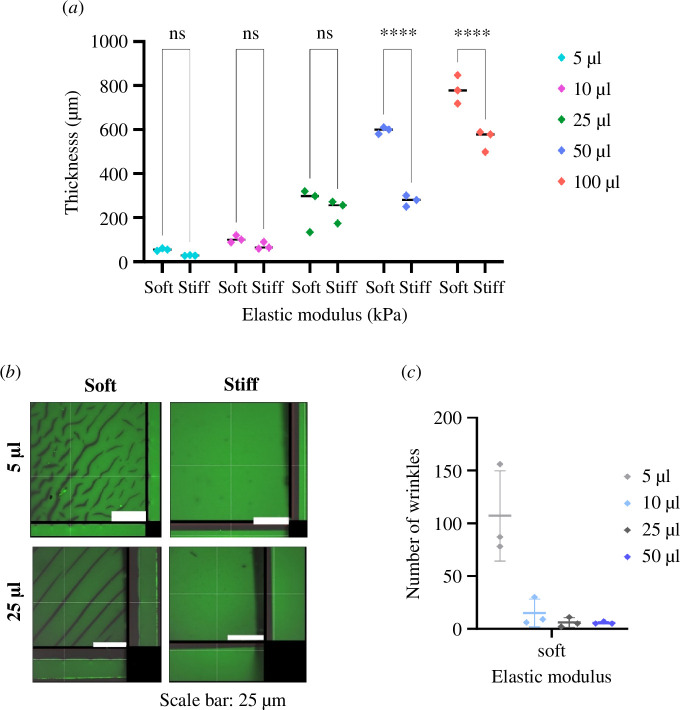
Hydrogel thickness is proportional to the PAAm mixture volume and influences the hydrogel surface and elastic modulus. (*a*) PAAm hydrogel thickness varies proportionally to the mixture volume, and soft hydrogels are thicker than their stiff counterparts at 50 µl. Thickness was measured by confocal microscopy. (*b*) Soft PAAm hydrogels exhibit surface wrinkles, whereas the surface of stiff PAAm hydrogels remains intact. Large images show *x*–*y* optical sections at the surface of the PAAm hydrogel marked with a line in the bottom and side *z*-images. (*c*) Wrinkles on soft PAAm hydrogels per field of view decrease relative to PAAm volume. A two-way ANOVA was used to test for significant differences (*n* = 3). ****p < 0.0001.

**Figure 2.**
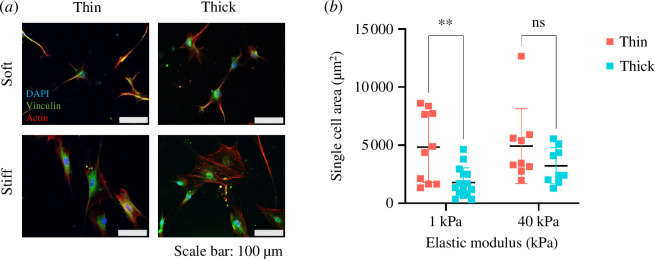
BMSCs mechanosense changes in elastic modulus and thickness and modify their spreading area and morphology in response. (*a*) Actin, nuclei and vinculin staining of BMSCs on PAAm hydrogels with different elastic modulus. Three pictures per hydrogel of each condition were taken and the number of cells varied. Short cells are appreciated on soft hydrogels, whereas more giant cells are seen on stiff hydrogels. No differences in actin or vinculin were appreciated. (*b*) Mean cell spreading area of BMSCs on soft and stiff, thin and thick PAAm hydrogels. BMSCs exhibit a greater cell spreading area on stiff, thin (9 cells) and soft, thin (10 cells) PAAm hydrogels. A Student’s *t*‐test was done to calculate significant differences of differences of means.

**Figure 3.**
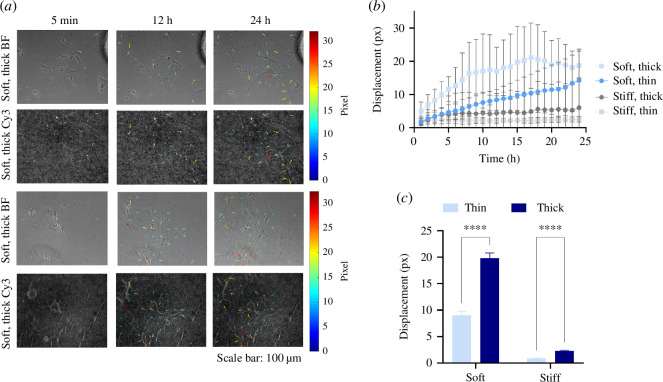
Hydrogel deformations by BMSCs increase over time and depend on the hydrogel’s mechanical properties. (*a*) Time-lapse imaging of the Stro-1+BMSCs on soft, thin and thick PAAm hydrogels with embedded fluorescent microbeads. Cell movements promote wrinkle formation over time; displacements indicated with coloured arrows. Note that arrow length was scaled by a factor of 3 for visualization. Phase contrast (BF) and fluorescent (Cy3) images were obtained at 10× magnification under a Nikon Eclipse Ti inverted microscope. (*b*) Hydrogel displacements increase over time and are greater on soft, thick hydrogels than on stiff matrices. Squares represent the mean and SD of the 90th percentiles of displacements of the 99 nodes (*n* = 3 hydrogels). (*c*) Stro-1+BMSCs generated more significant deformations on thick versus thin PAAm hydrogels, regardless of the hydrogel rigidity. A two-way ANOVA was used to assess the statistical significance of difference of means; ****p < 0.0001.

### 2.8. Computational model

A simple computational model was used to predict how the stiffness experienced by cells during contraction would depend on hydrogel thickness and lateral constraints (refer to electronic supplementary material, data 1).

### 2.9. Osteogenic differentiation, alkaline phosphatase activity and alizarin red S quantification

Cells were plated at 5000 cells cm^−2^ on tissue culture plastic (TCP) and PAAm hydrogels. One day post-seeding ascorbate-2-phosphate, beta glycerophosphate and dexamethasone were added to basal medium final concentrations of 280 µM, 5 mM and 10 nM, respectively. Medium was changed every 2 or 3 days for 7 or 14 days, before alkaline phosphatase (ALP) or alizarin red S biochemical quantification. A basal medium was used as a negative control. For ALP staining, medium was removed from the cells on TCP or PAAm hydrogels on day 7 or 14 and washed twice with PBS. Ethanol (95% v/v) was added to each well and incubated for 10 min at 4°C. Ethanol was aspirated, and the plate was washed twice with PBS before drying at room temperature. Fast violet (Sigma-Aldrich) (0.24 M) was dissolved in α-naftol solution in dH_2_O (4% v/v), added to the cells and incubated for 1 h at 37°C before removing the solution. The wells were rinsed with 1 ml Milli-Q water and imaged using a Zeiss microscope with a colour camera included (Axiovision software). For ALP quantification, medium was removed from the wells before adding 500 µl cell Lytic (Sigma-Aldrich) and incubated for 15 min. Later, the suspension was recovered and centrifuged at 4°C at 80*g* for 15 min. Finally, the cell suspension was recovered and transferred to new tubes, frozen at −80°C until their use. For the ALP quantification, the assay buffer for the standards and the ALP substrate solution (0.04 g phosphatase substrate (Sigma-Aldrich) in 10 ml alkaline buffer solution (1.5 M) (Sigma-Aldrich)). Later, *p*-nitrophenol standards at different concentrations were prepared. For the assay, 100 µl standards, 20 µl of cell lysate and 80 µl substrate were added in triplicates to a 96 transparent well plate. Additionally, 100 µl NaOH 1 M was added to each background control. After that, the well plate was incubated at 37°C for 60 min or until samples acquired a yellow colour, and the reaction was finished with 100 µl NaOH. The absorbance was quantified in a Glomax reader at 405 nm. Similarly, for alizarin red staining, medium was removed from the cells on TCP or PAAm hydrogels on day 14 and washed twice with PBS. The cells were then fixed with PFA (4% w/v) at room temperature for 15 min. Subsequently, PFA was aspirated and the cells were washed three times with deionized water. Later, 1 ml of filtered alizarin red staining solution (40 mM pH 4.5) (Sigma-Aldrich) was added to each well and left to incubate on the shaker at 180 r.p.m. for 1 h at room temperature. Finally, the solution was discarded, and the cells were rinsed with deionized water until no more stain was released. For alizarin red staining quantification, images were taken using a Zeiss microscope equipped with a colour camera (Axiovision software), and mean intensity was measured using CellProfiler.

## 3. Results

### 3.1. Control of polyacrylamide geometry and stiffness

To test the effect of hydrogel elastic modulus and thickness on the morphology, differentiation and cell-generated matrix displacements of BMSCs, we first prepared PAAm hydrogel substrates suitable for cell culture. The bulk modulus and thickness of substrates were controlled by varying the cross-linker and monomer concentration and volume solution, respectively, to produce ‘soft’ and ‘stiff’ hydrogels (of predicted elastic modulus 1 and 40 kPa, respectively) [2,13]. The thickness of both ‘soft’ and ‘stiff’ hydrogels increased as a function of the volume of polymer reaction solution used to form the gels, from 54.5 ± 5.7 to 597 ± 15 µm for soft hydrogel and from 27.4 ± 1.6 to 277 ± 25 µm for stiff hydrogels (figure 1*a*). The thickness of soft hydrogels was significantly greater than that of stiff hydrogels when made at a polymerization solution volume of 50 or 100 µl but not at any other volume. However, there was a general trend for stiff hydrogels to be thinner than soft hydrogels. Close examination of hydrogels by confocal sectioning revealed the presence of surface wrinkles on soft hydrogels, which were absent on stiff hydrogels (figure 1*b*). Wrinkles on thin materials were more tortuous and shorter than on thicker materials; in the latter, continuous wrinkles often extended over significant parts of the hydrogel surface. Reflecting this, the number of discrete wrinkles declined due to increasing thickness (figure 1*c*). In parallel, we used nanoindentation to measure substrate stiffness including a correction for hydrogel thickness [28] (electronic supplementary material, figure S1*c*,*d*). For ‘soft’ hydrogels formed from polymerization volumes greater than or equal to 25 µl corresponding to thicknesses greater than 200 µm, there were no significant differences in corrected elastic modulus, with mean values of 5.50 ± 2.0, 5.27 ± 1.5 and 5.51 ± 0.17 kPa for hydrogels formed from 25, 50 and 100 µl, respectively. However, for thin hydrogels made from 5 µl (approx. 50 µm), the corrected elastic modulus was slightly but significantly higher than for any other at 6.95 ± 0.15 kPa (*p* < 0.001). As expected, the corrected elastic modulus values for ‘stiff’ hydrogels were much greater than those for ‘soft’ hydrogels but with no significant difference between any group with values of 55.7 ± 0.89, 56.2 ± 0.42, 53.69 ± 1.41 and 59.77 ± 0.74 kPa for gels made from 5, 25, 50 and 100 µl gel solution, respectively. In subsequent experiments, PAAm hydrogels formed from 5 µl (‘thin’) or 25 µl (‘thick’) were used for all comparisons.

### 3.2. Hydrogel elastic modulus and thickness modify the spreading cell area of bone marrow stromal cells

In previous work by ourselves [13] and others [5], increasing stiffness leads to cell morphology and spreading changes. To confirm this, we plated primary BMSCs on the fabricated substrates and quantified cell morphology by microscopy. Cells stained with labelled phalloidin and immunostained for vinculin on stiff substrates approximately 200 µm in thickness appeared larger in area than those on soft materials, with more prominent pseudopodia (figure 2*a*). This was confirmed by quantifying cell area. Cells on stiff substrates had larger spreading areas (3230 ± 160 μm^2^) compared with cells on soft (1780 ± 1260 μm^2^) hydrogels (figure 2*b*, *p* < 0.05). Previous studies by Buxboim *et al.* [7] and Tusan *et al.* [13] found that substrate thickness dictates the apparent stiffness sensed by cells. To test this, we compared cell morphology and spreading on ‘soft’ hydrogels of thickness approximately 50 µm compared with approximately 200 µm. Cells on thin materials exhibited greater cell spreading area observed by microscopy (figure 2*c*), an observation confirmed by quantification of cell area, where cells on thin hydrogels spread to a greater degree (4840 ± 3040 μm^2^) compared with cells on thick hydrogels (1780 ± 1260 μm^2^; figure 2*d*). In addition, vinculin staining appeared qualitatively stronger and more extensive. Although cells on stiff materials or soft, thin substrates had significantly larger areas, the data tended to group into two populations, reflecting heterogeneity in cell response.

These data show that MSCs respond to increased detected substrate stiffness by increased spreading, either because of intrinsic bulk substrate elastic modulus or reduced substrate thickness.

### 3.3. Reduced substrate thickness restricts cell-induced substrate displacements

Increased cell spreading as a function of decreasing substrate thickness is probably due to the underlying glass surface’s constraint of cell-imparted hydrogel displacements. The cell interprets this as an increased material stiffness, despite the bulk modulus of the soft hydrogels remaining unchanged [8]. To test this hypothesis, we compared cell-induced displacements at the hydrogel surface of thin and thick, soft and stiff hydrogels by time-lapse displacement microscopy (note that this is an equivalent method to traction force microscopy, but in this study, we did not compute traction forces).

To achieve this, it was first necessary to include fluorescent particles in the hydrogels as fiduciary markers for digital image correlation analysis of time-lapse images (electronic supplementary material, figure S1*a*). Including these particles did not alter the thickness of the PAAm hydrogels, except for a volume of 50 µl hydrogel solution (electronic supplementary material, figure S1*b*). Fluorescent particles appeared to accumulate in areas of gel wrinkles and were observable as defined lines in fluorescent images of the gels. The addition of fluorescent particles caused minor changes in gel stiffness relative to controls, but did not affect the relative stiffness of ‘soft’ compared with ‘stiff’ hydrogels (electronic supplementary material, figure S1*c*,*d*).

Next, we plated BMSCs on thin (5 µl, approx. 50 µm) or thick (25 µl, approx. 200 µm) soft hydrogels and quantified cell-induced displacements over 24 h. Five minutes after cell seeding, BMSC morphology was similar regardless of the hydrogel’s elastic modulus or thickness (figure 3*a* and phase contrast videos in electronic supplementary material, video S1). However, it was evident from video microscopy of the fluorescence particle-labelled hydrogels that cells induced much larger displacements on thick substrates than those on thin substrates (electronic supplementary material, video S1). Note that aggregations of fluorescent particles were evident in areas where cells were not present, as well as in areas where they were, the former reflecting pre-cell seeding hydrogel swelling and the latter reflecting cell-induced gel contraction occurring before the start of video microscopy. To test the hypothesis that cells induce larger displacements on thick compared with thin substrates quantitatively, we measured hydrogel displacements and compared them between substrates. Hydrogel displacements increased over time in all cases, rising more quickly and to higher values on soft versus stiff materials and thick versus thin materials (figure 3*b*). When comparing cumulative displacements over the 24-time period, displacements were significantly greater for soft, thick substrates than soft, thin substrates (figure 3*c*). Despite the very low observable deformations, this trend was also apparent for stiff substrates (images and videos for stiff materials are available in electronic supplementary materials, figure S3 and video S2).

These data confirm that the surface displacements induced in soft hydrogels by BMSCs are reduced by decreasing hydrogel thickness due to constraints imposed by the proximity of the underlying glass surface to which the hydrogel is attached.

This is supported by the results of the computational model (electronic supplementary material, data 1), showing that stiffness increases sharply as hydrogel thickness is reduced, which would imply lower displacements for the same traction forces.

### 3.4. The osteogenic differentiation of bone marrow stromal cells does not depend on material elasticity and thickness

**Figure 4.**
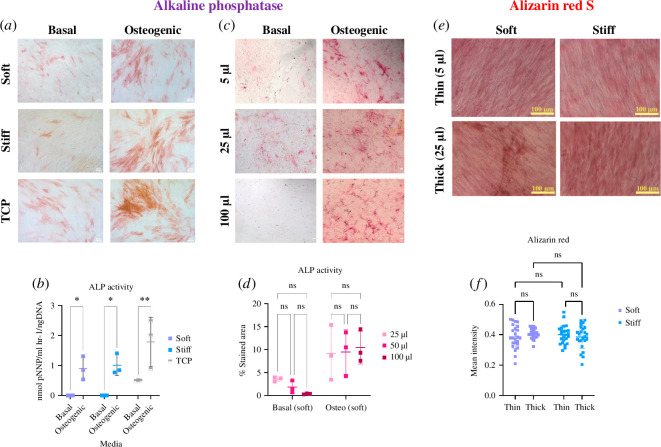
Substrate stiffness and thickness do not increase the osteogenic differentiation of BMSCs. (*a*) ALP staining of BMSCs on soft, stiff PAAm hydrogels and TCP in basal and osteogenic media. Osteogenic supplements but not substrate stiffness enhance the ALP activity of MSCs. Five images per hydrogel at each elastic modulus/thickness and TCP were taken using a Zeiss Axiovert 200 microscope. (*b*) ALP activity increased under osteogenic conditions compared with basal media; non-significant changes were detected with increasing stiffness. Squares represent the mean ALP activity normalized to DNA content and standard deviation. ALP activity was quantified on three hydrogels at each thickness/elastic modulus. Significant differences were calculated with the two-way ANOVA method. * *p* < 0.05 and ** p < 0.01. (*c*) ALP staining of MSCs on soft, stiff (thin and thick) PAAm hydrogels and TCP (*n* = 3) under osteogenic conditions. ALP staining increased on stiff compared with soft PAAm hydrogels, but no clear differences in thickness are seen (*d*) The addition of osteogenic supplements but not the change in substrate thickness increases the ALP activity of cells. Neither stiffness nor thickness had a significant effect on the deposition of Ca^2+^ as measured using alizarin red S, staining shown in (*e*) and quantification in (*f*). Squares represent the mean and s.d. of the percentage of the stained area. ALP activity and ARS staining were quantified on three hydrogels at each thickness/elastic modulus. The two-way ANOVA method was used to calculate significant differences.

BMSCs are known to differentiate into functionally distinct lineages due to changes in substrate elastic modulus, with increased differentiation into osteoblastic lineage cells on stiffer compared with softer materials [5]. To test whether BMSCs would perceive a reduction in the thickness of a material with a low elastic modulus as an increase in stiffness, we plated BMSCs on thick or thin substrates of high and low elastic modulus and measured osteogenic differentiation. As expected, osteogenic supplements induced the differentiation of primary BMSCs to the osteogenic lineage, as measured by alkaline phosphatase activity (figure 4*a*) or alizarin red S (electronic supplementary material, figure S2). However, in contrast to our hypothesis, we found no significant differences in differentiation because of increased bulk elastic modulus or because of decreased substrate thickness for alkaline phosphatase (figure 4*c,d*) or alizarin red S (figure 4*e*,*f*).

### 3.5. Cell-induced displacements decrease with respect to cell density

**Figure 5.**
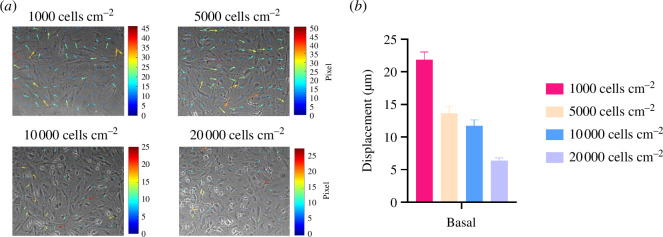
Matrix deformations decrease with respect to increased BMSCs seeding density. (*a*) Time-lapse imaging of MSCs at different seeding densities on soft, thick hydrogels (*n* = 3 per cell seeding density tested) in basal media at 5 min and 20 h. Coloured arrows indicate the increase in hydrogel deformations. Scale arrow = 3. Phase contrast (BF) images were obtained at 10× magnification under a Nikon Eclipse Ti inverted microscope; one field per soft/thick hydrogel (*n* = 3 at each seeding density) was taken. (*b*) BMSCs created greater hydrogel deformations at lower seeding densities after 20 h of incubation in basal conditions. Bars represent the mean and s.d. of the 90th percentiles of the hydrogel displacements (*n* = 3 hydrogels per seeding density tested) were quantified hourly and analysed for the last 11 h of the experiment. Significant differences were found between all groups using the two-way ANOVA method with ****p < 0.001 between 1000 cell/cm² and 20,000 cells/cm².

One explanation for the lack of differentiation in marrow stromal cells as a function of stiffness or thickness may be constraints imposed by neighbouring cells at high density. As reported in a previous study [27], this effect has been hypothesized to reduce the ability of cells to mechanosense soft materials due to competing tractions from neighbouring cells [17]. In addition, large cumulative tractions induced on substrates by monolayers of cells may result in insensitivity to substrate elastic modulus due to whole-scale contraction of the hydrogel by collective cell action [13]. To directly test this, we plated BMSCs at increasing densities on soft, thick materials (283 ± 50 µm) and quantified hydrogel displacements at 20 h. As predicted by Venugopal *et al*. [27], displacements declined as a function of cell density (figure 5*a,b* and electronic supplementary material, video S3). Cells at 1000 cells cm^2^ created greater hydrogel displacements (21.8 ± 1.1 µm) compared with the displacements generated by cells at 20 000 cells cm^2^ (6.3 ± 0.4 µm).

### 3.6. Cell-induced displacements decrease with respect to time of culture

**Figure 6.**
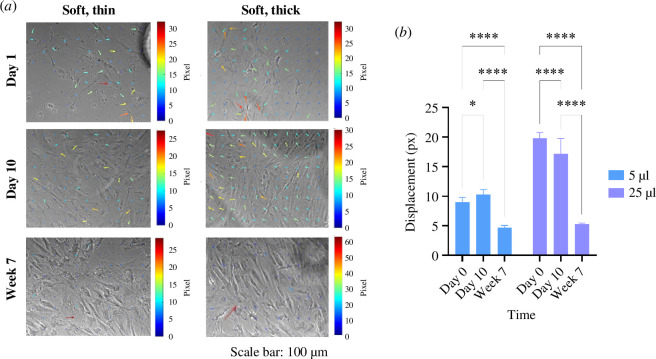
BMSC-induced hydrogel deformations decrease with respect to culture time. (*a*) Time-lapse imaging of cells on soft, thin and thick hydrogels on day 1, day 10 and week 7 after 24 h of incubation. During cell spreading, cells create more wrinkles on soft, thick hydrogels than on soft, thin counterparts, indicated with coloured arrows on day 1; increased cell crowding is appreciated on day 10; and coloured arrows indicate more hydrogel deformations on soft, thick PAAm hydrogels; cell crowding definitely increase on week 7, hindering hydrogel deformations. Phase contrast images were obtained at 10× magnification under a Nikon Eclipse Ti inverted microscope. Scale arrow = 3. One microscopic field per soft, thin and soft, thick hydrogel (*n* = 3) was imaged. (*b*) Soft, thick hydrogels suffer greater deformations than soft, thin counterparts by MSCs that decrease over time from day 1 to week 7. Bars represent the mean and standard deviation of the 90th percentiles of the hydrogel displacements on soft, thin and thick materials on day 1, day 10 and week 7 during the last 12 h of the experiment.

As osteogenic differentiation intrinsically relies on cell–cell contact and extended culture, we hypothesized that cell crowding during prolonged cell culture might abrogate cell mechanosensing by collective cell behaviour. To test this, we plated BMSCs at 5000 cells/cm^2^ on thin and thick soft substrates in basal medium and tracked displacements every 5 min at 24 h, 10 days and 7 weeks post-seeding. At early time points (24 h), hydrogel deformations were greater for cells on thick PAAm hydrogels than thin hydrogels (figure 6*a,b*; also observed in electronic supplementary material, video S4). As expected, cell density on all hydrogels increased after 10 days or 7 weeks compared with day 1, as observed in the representative pictures in figure 6*a*. At 10 days, there were still significantly greater displacements measured on thick compared with thin gels; however, by 7 weeks, there was no significant difference (*p* < 0.05). In parallel, there was a significant decrease in mean displacements for cells on thick hydrogels concerning time between day 10 and week 7 (figure 6*b*; 19.8 ± 0.9 µm on day 1 compared with 5.2 ± 0.1 µm on week 7 (thick hydrogels); 8.9 ± 0.8 µm on day 1 compared with 4.7 ± 0.3 µm on week 7 (thin hydrogels); *p* < 0.0001).

**Figure 7.**
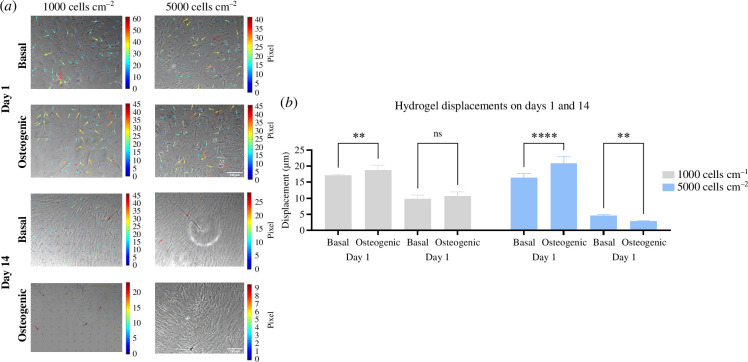
BMSCs generated different hydrogel deformations in basal and osteogenic conditions at different seeding densities. (*a*) Time-lapse images of MSCs on soft, thick PAAm hydrogels in basal and osteogenic media at low seeding densities (1000 and 5000 cells cm^−2^) on days 1 and 14. Cell morphology and alignment varied with the increase in seeding density. Coloured arrows show displacements on soft, thick hydrogels on day 1 but not on day 14. Images were obtained at 10× magnification with a Nikon Eclipse Ti inverted microscope. Scale arrow = 3. One microscopic field per hydrogel (*n* = 3) at each condition was imaged. (*b*) Hydrogel displacements by MSCs decreased on day 14 compared with day 1, regardless of the seeding density. Deformations slightly increased in osteogenic conditions compared with basal media at 1000 cells cm^−2^ but increased in basal compared with osteogenic media at 5000 cells cm^−2^ after 14 days of incubation. Bars represent the mean and standard deviation of the 90th percentiles of the hydrogel (*n* = 3) displacements on soft, thick materials on days 1 and 14 in basal and osteogenic conditions during the last 11 h of the experiment. ** desinates p < 0.01 and **** p < 0.0001.

We also compared time-dependent cell-induced displacements concerning time in basal and osteogenic medium. For low and moderate cell densities, there were significant increases in cell contractility in cells plated in the osteogenic medium after only 24 h (figure 7*a,b* and electronic supplementary material, video S5). As in other experiments, cell displacements’ magnitude declined over time, with a greater decline in cells plated at higher density. This effect was more pronounced in cells in osteogenic conditions, where displacements were significantly reduced compared with basal (3.6 ± 0.5 µm versus 7.1 ± 0.6 µm).

These data indicate a time-dependent decrease in cell-induced hydrogel displacements for cells on thick materials, which is associated with an increase in cell density and the addition of osteogenic medium.

## 4. Discussion

The elastic modulus of ECM and biomaterials is known to be a factor in directing the differentiation of BMSCs, with materials of higher modulus promoting osteogenic differentiation. However, material geometry affects the true stiffness that individual and groups of cells ‘feel’. In this study, we found that matrix deformations exerted by BMSCs are constrained by both material thickness and cell density, which may provide a mechanism for why in some circumstances BMSCs become insensitive to substrate stiffness during differentiation to the osteoblastic lineage.

We first observed that soft hydrogels made from equivalent concentrations of monomer and cross-linker were thicker than their stiff counterparts. This is probably due to swelling. Softer hydrogels have been shown to have larger pores than stiff hydrogels, and hold more water molecules. Subramani *et al.* and Protick *et al.* [31,32] showed that the swelling ratio decreased on stiffer hydrogels, approximately 900% for soft hydrogels and approximately 350% for stiff hydrogels. Although there was a general trend in this effect for hydrogels, the effect was most pronounced at 50 µl, where the gel is less constrained by the underlying hydrogel, enabling it to swell to a greater extent. In parallel with swelling, we also found that soft hydrogels exhibited wrinkles on their surfaces. This agrees with a previous report showing that during soft hydrogel fabrication, wrinkles appear due to the differences in osmotic pressure in buffer or media solutions, causing water ingress and thus swelling [33]. We noticed that the number of wrinkles decreased and that their length increased as a function of increasing hydrogel thickness. As gels are covalently coupled to the underlying glass support, the hydrogel can only swell in a direction apical to the glass surface, resulting in a compressive stress in the hydrogel layer which may then induce buckling at the surface of the hydrogel. Regardless of these differences, we found previously that these hydrogels differ considerably in their stiffness [28], and exhibit large, flat areas easily distinguishable from wrinkled areas, making them suitable for quantitative cell culture studies, although we recognize that wrinkles may be a confounding factor in studies of differentiation where effects are averaged across a culture well.

The observation that BMSCs respond to materials of different elastic modulus by modulating the degree of spreading and actin fibre generation is well understood [34,35]. Cells on materials of a low elastic modulus are unable to generate cytoskeletal tension and generally appear smaller. In contrast, cells on materials with higher moduli generate higher forces that promote the formation of rigid, stiff and contractile stress fibres, which promote cell spreading [2,36]. In addition, our data showing that cells spread to a greater extent on thick, soft materials compared with thin ones also reflects extensive literature supporting the ability of cells to detect boundaries [7,10,37]. It has previously been assumed that this is probably due to constraint to cell-induced lateral hydrogel deformation imposed by an underlying stiff support [10]. Our data showing reduced displacements on materials of identical composition but different thicknesses indicate that this unexplored hypothesis is probably correct and reflect our earlier data showing that groups of osteosarcoma cells impose smaller displacements on thin compared with thick hydrogels [13].

Numerous previous studies reported that changes in ECM stiffness influence the differentiation potential of BMSCs (also sometimes known as MSCs) [5,38]. ECM stiffness is know to affect stem cell differentiation to the specific cell type that matches the tissue stiffness: 0.1–1 kPa hydrogels are neurogenic, 8–17 kPa are myogenic and 25–40 kPa are osteogenic [5]. We evaluated the osteogenic differentiation potential of BMSCs by quantifying ALP activity, a marker for BMSC osteogenic differentiation. Despite other data reporting that stiff hydrogels promote an increase in cell proliferation and osteogenic differentiation, we found no significant difference in ALP activity between soft and stiff hydrogels with different thicknesses. Previous reports indicate that high seeding density may abrogate the ability of cells to detect soft materials [27].

These results led us to quantify hydrogel displacements to test whether cell-induced hydrogel displacements were inhibited with respect to cell density. We observed that cells created greater deformations on soft, thick hydrogel deformations at low seeding density compared with high seeding density in both basal and osteogenic medium. This probably reflects inhibition of cell contractility due to a ‘tug-of-war’ between neighbouring cells [12], with cells mechanically coupled both by direct contact or through the underlying material [17]. We suspect that in subconfluent monolayers cells BMSCs may in fact be mechanically ‘coupled’ across the entirety of the hydrogel with the underlying glass support, detecting a higher stiffness than the independently measured modulus of the material might suggest. Computational modelling (electronic supplementary material, data 1) supports the notion that cells subject to lateral constraints as imposed by neighbouring cells experience increased stiffness, but that this may only emerge at relatively high density. Further experiments that may include measurements of whole-gel contraction, or control of the lateral dimensions of BMSCs layers, would be required to test this hypothesis formally. In addition, it is very challenging to test the effect of cell density-dependent, dynamic matrix displacements as a causative factor in differentiation due to confounding variables such as direct cell–cell contact or changes in paracrine signalling. In future work, it may be possible to control this by, for example, inducing mechanical tension in the gel periodically or continuously to mimic the gel displacements proximal cells might induce.

In most experimental protocols, terminal osteogenic differentiation of BMSCs usually requires prolonged cell culture (two to three weeks or more) and cell–cell contact. The observation that displacements also declined with respect to culture time (which is positively correlated with cell density) also supports this hypothesis. However, it is challenging here to rule out any effect of ECM deposition on the surface of the PAAm. It may be the case that at later time points, cells have secreted ECM which provides a stiffer growth substratum for the cells and which prevents direct mechanical coupling between cells and the underlying (fiduciary marker labelled) PAAm [39]. Studies over prolonged periods of time may also lead to some hydrolysis of PAAm gels, although this has not been found to lead to any material degradation [40]. Future studies may address directly the impact of cell culture conditions on PAAm gels with respect to time. We chose PAAm due to its linear elasticity, isotropy, ease of handling and compatibility with displacement tracking microscopy. While this makes it a useful material for studying fundamental principles in mechanobiology research, we recognize it does not reflect the complex nonlinear properties of native ECM and tissues. Fibrous matrices like collagen are viscoelastic, and cell-induced deformations induce anisotropic reorganization of its constituent fibres. This may result in strain-dependent changes in the stiffness that cells detect, a situation that becomes more complex for large strains imposed by groups of cells. It is clear from both modelling [41] and experimental work [42] that gels on or inside collagen gels are sensitive to material thickness, but that the anisotropy of the collagen material modifies the range at which stiffness sensing occurs. This underscores that the effects we observe in the current study are likely to hold true for other materials, but with different relationships between cell number and material thickness, for example.

Further to these data, we found that BMSCs exerted significantly greater surface displacements when plated in medium containing osteogenic supplements than in basal medium. We consider that this is probably due to the glucocorticoid, dexamethasone, which has been shown to increase cell contractility in a range of cells, including MSCs [43] and alveolar epithelial cells [44], possibly through a role in modulating the formation and stability of F-actin [45]. In addition, it was also evident that displacements were either unchanged or significantly lower for cells cultured in osteogenic medium compared with basal medium at later time points. It is likely that this was due to increased proliferation in osteogenic conditions [46], leading to higher cell density and reduced measured displacements, by the mechanism we propose above.

In summary, cell mechanosensing is a complex process involving different variables such as ECM modulus and thickness, cell density and shape and the presence of supplements, determining cell-induced hydrogel displacements and cell differentiation.

## Data Availability

All supplementary files including videos and code are available on request from the authors or can be found at [47]. Supplementary material is available online [48].

## References

[1] Ringer P , Colo G , Fässler R , Grashoff C . 2017 Sensing the mechano-chemical properties of the extracellular matrix. Matrix Biol. **64** , 6–16. (10.1016/j.matbio.2017.03.004) 28389162

[2] Pelham RJ , Wang Y l . 1997 Cell locomotion and focal adhesions are regulated by substrate flexibility. Proc. Natl Acad. Sci. USA **94** , 13 661–13 665. (10.1073/pnas.94.25.13661) PMC28362 9391082

[3] Lo CM , Wang HB , Dembo M , Wang YL . 2000 Cell movement is guided by the rigidity of the substrate. Biophys. J. **79** , 144–152. (10.1016/S0006-3495(00)76279-5) 10866943 PMC1300921

[4] Wang Y , Wang G , Luo X , Qiu J , Tang C . 2012 Substrate stiffness regulates the proliferation, migration, and differentiation of epidermal cells. Burns **38** , 414–420. (10.1016/j.burns.2011.09.002) 22037151

[5] Engler AJ , Sen S , Sweeney HL , Discher DE . 2006 Matrix elasticity directs stem cell lineage specification. Cell **126** , 677–689. (10.1016/j.cell.2006.06.044) 16923388

[6] Evans ND , Minelli C , Gentleman E , LaPointe V , Patankar SN , Kallivretaki M , Chen X , Roberts CJ , Stevens MM . 2009 Substrate stiffness affects early differentiation events in embryonic stem cells. Eur. Cell. Mater. **18** , 1–13; (10.22203/ecm.v018a01) 19768669

[7] Buxboim A , Rajagopal K , Brown AEX , Discher DE . 2010 How deeply cells feel: methods for thin gels. J. Phys. Condens. Matter **22** , 194116. (10.1088/0953-8984/22/19/194116) 20454525 PMC2864502

[8] Evans ND , Gentleman E . 2014 The role of material structure and mechanical properties in cell–matrix interactions. J. Mater. Chem. B **2** , 2345–2356. (10.1039/c3tb21604g) 32261407

[9] Swift J *et al* . 2013 Nuclear lamin-A scales with tissue stiffness and enhances matrix-directed differentiation. Science **341** , 1240104. (10.1126/science.1240104) 23990565 PMC3976548

[10] Leong WS , Tay CY , Yu H , Li A , Wu SC , Duc DH , Lim CT , Tan LP . 2010 Thickness sensing of hMSCs on collagen gel directs stem cell fate. Biochem. Biophys. Res. Commun. **401** , 287–292. (10.1016/j.bbrc.2010.09.052) 20851103

[11] Lin YC , Tambe DT , Park CY , Wasserman MR , Trepat X , Krishnan R , Lenormand G , Fredberg JJ , Butler JP . 2010 Mechanosensing of substrate thickness. Phys. Rev. E. Stat. Nonlin. Soft Matter Phys. **82** , 041918. (10.1103/PhysRevE.82.041918) 21230324 PMC3641827

[12] Trepat X , Wasserman MR , Angelini TE , Millet E , Weitz DA , Butler JP , Fredberg JJ . 2009 Physical forces during collective cell migration. Nat. Phys. **5** , 426–430. (10.1038/nphys1269)

[13] Tusan CG *et al* . 2018 Collective cell behavior in mechanosensing of substrate thickness. Biophys. J. **114** , 2743–2755. (10.1016/j.bpj.2018.03.037) 29874622 PMC6027966

[14] Zarkoob H , Bodduluri S , Ponnaluri SV , Selby JC , Sander EA . 2015 Substrate stiffness affects human keratinocyte colony formation. Cell. Mol. Bioeng. **8** , 32–50. (10.1007/s12195-015-0377-8) 26019727 PMC4442095

[15] Mertz AF , Banerjee S , Che Y , German GK , Xu Y , Hyland C , Marchetti MC , Horsley V , Dufresne ER . 2012 Scaling of traction forces with the size of cohesive cell colonies. Phys. Rev. Lett. **108** , 198101. (10.1103/PhysRevLett.108.198101) 23003091 PMC4098718

[16] Emerman JT , Pitelka DR . 1977 Maintenance and induction of morphological differentiation in dissociated mammary epithelium on floating collagen membranes. In Vitro **13** , 316–328. (10.1007/BF02616178) 559643

[17] Reinhart-King CA , Dembo M , Hammer DA . 2008 Cell-cell mechanical communication through compliant substrates. Biophys. J. **95** , 6044–6051. (10.1529/biophysj.107.127662) 18775964 PMC2599854

[18] El-Rashidy AA , El Moshy S , Radwan IA , Rady D , Abbass MMS , Dörfer CE , Fawzy El-Sayed KM . 2021 Effect of polymeric matrix stiffness on osteogenic differentiation of mesenchymal stem/progenitor cells: concise review. Polymers **13** , 2950. (10.3390/polym13172950) 34502988 PMC8434088

[19] Wen JH , Vincent LG , Fuhrmann A , Choi YS , Hribar KC , Taylor-Weiner H , Chen S , Engler AJ . 2014 Interplay of matrix stiffness and protein tethering in stem cell differentiation. Nat. Mater. **13** , 979–987. (10.1038/nmat4051) 25108614 PMC4172528

[20] Lee J , Abdeen AA , Huang TH , Kilian KA . 2014 Controlling cell geometry on substrates of variable stiffness can tune the degree of osteogenesis in human mesenchymal stem cells. J. Mech. Behav. Biomed. Mater. **38** , 209–218. (10.1016/j.jmbbm.2014.01.009) 24556045

[21] Witkowska-Zimny M , Walenko K , Wrobel E , Mrowka P , Mikulska A , Przybylski J . 2013 Effect of substrate stiffness on the osteogenic differentiation of bone marrow stem cells and bone-derived cells. Cell Biol. Int. **37** , 608–616. (10.1002/cbin.10078) 23447501

[22] Rowlands AS , George PA , Cooper-White JJ . 2008 Directing osteogenic and myogenic differentiation of MSCs: interplay of stiffness and adhesive ligand presentation. Am. J. Physiol., Cell Physiol. **295** , C1037–44. (10.1152/ajpcell.67.2008) 18753317

[23] Barreto S , Gonzalez-Vazquez A , Cameron AR , Cavanagh B , Murray DJ , O’Brien FJ . 2017 Identification of the mechanisms by which age alters the mechanosensitivity of mesenchymal stromal cells on substrates of differing stiffness: implications for osteogenesis and angiogenesis. Acta Biomater. **53** , 59–69. (10.1016/j.actbio.2017.02.031) 28216301

[24] Viale-Bouroncle S , Völlner F , Möhl C , Küpper K , Brockhoff G , Reichert TE , Schmalz G , Morsczeck C . 2011 Soft matrix supports osteogenic differentiation of human dental follicle cells. Biochem. Biophys. Res. Commun. **410** , 587–592. (10.1016/j.bbrc.2011.06.031) 21684253

[25] Stanton AE , Tong X , Yang F . 2019 Extracellular matrix type modulates mechanotransduction of stem cells. Acta Biomater. **96** , 310–320. (10.1016/j.actbio.2019.06.048) 31255664 PMC8735670

[26] Mao AS , Shin JW , Mooney DJ . 2016 Effects of substrate stiffness and cell-cell contact on mesenchymal stem cell differentiation. Biomaterials **98** , 184–191. (10.1016/j.biomaterials.2016.05.004) 27203745 PMC4906313

[27] Venugopal B , Mogha P , Dhawan J , Majumder A . 2018 Cell density overrides the effect of substrate stiffness on human mesenchymal stem cells’ morphology and proliferation. Biomater. Sci. **6** , 1109–1119. (10.1039/c7bm00853h) 29528341 PMC5933002

[28] Xu D , Hernandez Miranda ML , Evans ND , Sengers BG , Browne M , Cook RB . 2023 Depth profiling via nanoindentation for characterisation of the elastic modulus and hydraulic properties of thin hydrogel layers. J. Mech. Behav. Biomed. Mater. **148** , 106195. (10.1016/j.jmbbm.2023.106195) 37862727

[29] Janeczek AA , Tare RS , Scarpa E , Moreno-Jimenez I , Rowland CA , Jenner D , Newman TA , Oreffo ROC , Evans ND . 2016 Transient canonical wnt stimulation enriches human bone marrow mononuclear cell isolates for osteoprogenitors. Stem Cells **34** , 418–430. (10.1002/stem.2241) 26573091 PMC4981914

[30] Kanczler J , Tare RS , Stumpf P , Noble TJ , Black C , Oreffo ROC . 2019 Isolation, differentiation, and characterization of human bone marrow stem cells in vitro and in vivo. Methods Mol. Biol. **1914** , 53–70. (10.1007/978-1-4939-8997-3_4) 30729460

[31] Subramani R , Izquierdo-Alvarez A , Bhattacharya P , Meerts M , Moldenaers P , Ramon H , Van Oosterwyck H . 2020 The influence of swelling on elastic properties of polyacrylamide hydrogels. Front. Mater. **7** , 7. (10.3389/fmats.2020.00212)

[32] Protick FK , Amit SK , Amar K , Nath SD , Akand R , Davis VA , Nilufar S , Chowdhury F . 2022 Additive manufacturing of viscoelastic polyacrylamide substrates for mechanosensing studies. ACS Omega **7** , 24 384–24 395. (10.1021/acsomega.2c01817) PMC9301700 35874232

[33] Saha K , Kim J , Irwin E , Yoon J , Momin F , Trujillo V , Schaffer DV , Healy KE , Hayward RC . 2010 Surface creasing instability of soft polyacrylamide cell culture substrates. Biophys. J. **99** , L94–96. (10.1016/j.bpj.2010.09.045) 21156124 PMC3000484

[34] Wang HB , Dembo M , Wang YL . 2000 Substrate flexibility regulates growth and apoptosis of normal but not transformed cells. Am. J. Physiol. Cell Physiol. **279** , C1345–50. (10.1152/ajpcell.2000.279.5.C1345) 11029281

[35] Yeung T *et al* . 2005 Effects of substrate stiffness on cell morphology, cytoskeletal structure, and adhesion. Cell Motil. Cytoskeleton **60** , 24–34. (10.1002/cm.20041) 15573414

[36] Zhou DW , Lee TT , Weng S , Fu J , García AJ . 2017 Effects of substrate stiffness and actomyosin contractility on coupling between force transmission and vinculin-paxillin recruitment at single focal adhesions. Mol. Biol. Cell **28** , 1901–1911. (10.1091/mbc.E17-02-0116) 28468976 PMC5541841

[37] Merkel R , Kirchgessner N , Cesa CM , Hoffmann B . 2007 Cell force microscopy on elastic layers of finite thickness. Biophys. J. **93** , 3314–3323. (10.1529/biophysj.107.111328) 17660320 PMC2025665

[38] Lanniel M , Huq E , Allen S , Buttery L , Williams PM , Alexander MR . 2011 Substrate induced differentiation of human mesenchymal stem cells on hydrogels with modified surface chemistry and controlled modulus. Soft Matter **7** , 6501. (10.1039/c1sm05167a)

[39] Li B , Moshfegh C , Lin Z , Albuschies J , Vogel V . 2013 Mesenchymal stem cells exploit extracellular matrix as mechanotransducer. Sci. Rep. **3** , 2425. (10.1038/srep02425) 23939587 PMC3741624

[40] Ilavsky M , Hrouz J , Stejskal J , Bouchal K . 1984 Phase transition in swollen gels. 6. Effect of aging on the extent of hydrolysis of aqueous polyacrylamide solutions and on the collapse of gels. Macromolecules **17** , 2868–2874. (10.1021/ma00142a072)

[41] Mullen CA , Vaughan TJ , Billiar KL , McNamara LM . 2015 The effect of substrate stiffness, thickness, and cross-linking density on osteogenic cell behavior. Biophys. J. **108** , 1604–1612. (10.1016/j.bpj.2015.02.022) 25863052 PMC4390808

[42] Unnikandam Veettil SR , Van Bruggen SM , Hwang DG , Bartlett MD , Schneider IC . 2019 Tuning surface functionalization and collagen gel thickness to regulate cancer cell migration. Colloids Surf. B. Biointerfaces **179** , 37–47. (10.1016/j.colsurfb.2019.03.031) 30933893

[43] Sridharan Weaver S , Li Y , Foucard L , Majeed H , Bhaduri B , Levine AJ , Kilian KA , Popescu G . 2019 Simultaneous cell traction and growth measurements using light. J. Biophotonics **12** , e201800182. (10.1002/jbio.201800182) 30105846 PMC7236521

[44] Puig F , Gavara N , Sunyer R , Carreras A , Farré R , Navajas D . 2009 Stiffening and contraction induced by dexamethasone in alveolar epithelial cells. Exp. Mech. **49** , 47–55. (10.1007/s11340-007-9072-6)

[45] Castellino F , Heuser J , Marchetti S , Bruno B , Luini A . 1992 Glucocorticoid stabilization of actin filaments: a possible mechanism for inhibition of corticotropin release. Proc. Natl Acad. Sci. USA **89** , 3775–3779. (10.1073/pnas.89.9.3775) 1315038 PMC525573

[46] Nishimura I , Hisanaga R , Sato T , Arano T , Nomoto S , Ikada Y , Yoshinari M . 2015 Effect of osteogenic differentiation medium on proliferation and differentiation of human mesenchymal stem cells in three-dimensional culture with radial flow bioreactor. Regen. Ther. **2** , 24–31. (10.1016/j.reth.2015.09.001) 31245456 PMC6581791

[47] Hernandez-Miranda ML , Xu D , Ben Issa AA , Johnston DA , Browne M , Cook RB , Sengers BG , Evans N . 2023 Supplementary videos of the thesis: Individual and collective mechanosensing of extracellular matrix thickness in skeletal stem cell differentiation. [Dataset] (10.5258/SOTON/D2640)

[48] Hernandez-Miranda ML , Xu D , Issa B , Aya A , Johnston DA , Browne M , Cook RB , Evans N . 2024 Data from: Geometric constraint of mechanosensing by modification of hydrogel thickness prevents stiffness-induced differentiation in bone marrow stromal cells. Figshare. (10.6084/m9.figshare.c.7454665) PMC11444768 39353563

